# Chiropractors’ perceptions on the use of spinal radiographs in clinical practice: a qualitative study

**DOI:** 10.1186/s12998-024-00547-y

**Published:** 2024-06-22

**Authors:** Isaac Searant, Benjamin T. Brown, Hazel J Jenkins

**Affiliations:** Department of Chiropractic, Faculty of Medicine, Health and Human Sciences, Macquarie University, Sydney, Australia

**Keywords:** Chiropractic, Radiography, Spine, Clinical decision-making

## Abstract

**Background:**

Radiography is commonly used in the assessment of spinal disorders, despite a lack of high-quality evidence demonstrating improved clinical outcomes or additional benefit to the patient. There is disagreement amongst chiropractors regarding the appropriate use of radiography for clinical management. This study aims to qualitatively explore chiropractors’ perceptions on the use of spinal radiographs in clinical practice with respect to how they determine when to order radiographs; and how they use radiographs to inform clinical management.

**Methods:**

Online qualitative semi-structured interviews were conducted with 17 Australian chiropractors who currently manage patients with spinal disorders. Convienence, snowball, and purposive sampling strategies were used to ensure an appropriate breadth and depth of participant characterisitcs and beliefs. Interview data were recorded, transcribed and analysed using framework analysis.

**Results:**

Three themes were developed to describe how chiropractors determined when to order radiographs. These themes included specific findings from the clinical encounter that may inform clinical management, their perceptions of radiation risk, and the influence of clinical experience/intuition. Three themes and four subthemes were developed for how chiropractors use radiographs to inform their management. These themes explored the use of radiography for the application of chiropractic technique, as well as the role of radiographs in predicting patient prognosis, and as an educational tool to provide reassurance.

**Conclusion:**

Australian chiropractors’ decision-making around spinal radiography is diverse and can be influenced by a number of clinical and external factors. Previously unexplored uses of spinal radiography in clinical practice were highlighted. Some chiropractors reported potential benefits of radiography that are currently not supported by research evidence. Future research should address how radiographic findings are reported to patients with spinal disorders and how this could be optimised to improve patient outcomes.

**Supplementary Information:**

The online version contains supplementary material available at 10.1186/s12998-024-00547-y.

## Background

Diagnostic imaging modalities such as radiography are commonly used in the assessment of spinal pain [1]. Approximately one in four people with a new episode of low back pain (LBP) are referred for imaging [2]. However, one third of diagnostic imaging for LBP may not be clinically indicated as per current primary care Clinical Practice Guidelines (CPGs) [3, 4]. Reasons for imaging referral that are CPG concordant include: the suspicion of serious pathology (fracture, cauda equina, cervical myelopathy, malignancy, inflammatory arthritis, and infection); non-response to a trial of conservative management; surgical candidates; and suspicion of a clinically significant scoliosis [5–7]. The use of imaging in scenarios where there is unclear clinical benefit is a significant burden to the healthcare systems via wasted resources [8], and may cause harm to patients [9]. In 2019, diagnostic imaging cost the Australian public healthcare system $272 million [10]. Potential harm to patients can include unnecessary radiation exposure [9], increased downstream costs to the patient [11], increased/unnecessary healthcare utilisation [8, 12], and potential over-diagnosis due to the unclear relationships between imaging findings and clinical symptoms [13–15].

Different health care practitioners can refer for spinal radiographs, including chiropractors. In a global workforce review of the profession, including Australia, chiropractors are permitted to own radiographic equipment and refer for spinal radiographs in 43/90 (47.8%) of countries where chiropractors practice [16]. The proportion of patients referred for imaging ranges widely, from 8 to 84% [9]. The wide range in reported usage may reflect differences in opinion between chiropractors regarding the appropriate use of radiographs in clinical practice. While some chiropractors primarily use radiographs to rule out serious pathology and trauma, which is in line with CPGs [9, 17], others consider such recommendations to be too restrictive. Some chiropractors suggest that radiographs are additionally necessary in the assessment of structural misalignment, to determine/guide appropriate treatment, and to identify clinically unsuspected contraindications to management [18, 19]. For example, a survey of United States (US) chiropractors reported that 87% of chiropractors thought obtaining radiographs for biomechanical analysis has significant value, and 72% of chiropractors reported using radiographs to determine where and how to perform spinal manipulation (SM) [19]. Similarly, in Australia, approximately one-third of chiropractors mentioned that they would refer for radiographs to perform biomechanical analyses, and approximately two-thirds stated that they would use radiographs to assess patients for less serious conditions (e.g., spinal curve changes, spondylolisthesis, or degeneration) [20]. Given the common use of SM by chiropractors, and potential risk of adverse events associated with the use of SM in patients with underlying pathology (e.g., fracture in osteoporotic patients), some chiropractors argue that alternative/revised radiographic guidelines are needed [21, 22].

Despite disagreement amongst chiropractors about the clinical use of radiography, no high-quality evidence currently exists demonstrating improved clinical outcomes or additional benefit to the patient when imaging is used by chiropractors in ways that fall outside of current CPG recommendations [9, 17, 23, 24]. Previous literature has explored barriers and facilitators around chiropractor’s adherence to radiographic guidelines [25–27]. However, there is a paucity of literature regarding how chiropractors use spinal radiography after they have referred for it, and whether they perceive that radiography makes a difference in their clinical management of spinal disorders.

This study aims to qualitatively explore chiropractors’ perceptions on the use of spinal radiographs in clinical practice with respect to:

1. How they determine when to order radiographs; and
2. How they use radiographs to inform clinical management

## Methods

### Study design

Semi-structured interviews were used to explore chiropractors’ perceptions of the use of spinal radiographs in clinical practice. Ethical approval was provided by Macquarie University Human Research Ethics Committee (Approval No.: 520,221,146,637,793). This study was reported in accordance with the Consolidated Criteria for Reporting Qualitative Research (COREQ) checklist [28].

### Participants

#### Recruitment

Any currently registered Australian chiropractors who reported managing spinal disorders were eligible for inclusion in the study. Participants were recruited through social media advertisements (Facebook, including Australian chiropractic Facebook groups), convenience sampling of the research teams’ professional networks, and via snowball sampling. Chiropractors who responded to calls for recruitment were asked to provide consent and complete a short questionnaire to collect demographic data: sex; years in practice; practice setting (rural/urban); state/territory of practice; chiropractic techniques used in practice, frequency of use of radiographs; place of radiography referral; and beliefs around the use of radiographs. Purposive sampling, to ensure a broad spread of chiropractor demographic characteristics, was then carried out based on the respondents’ answers, and participants were invited via email to take part in an interview. Participant recruitment was conducted concurrently with data collection and analysis in order to achieve appropriate breadth and depth of participant data [29].

#### Interviews

Semi-structured interviews were conducted between June and August 2022 by IS. All interviews were conducted via Microsoft Teams and were audio recorded. A pilot interview was conducted by IS with a chiropractor not involved in the study to test the screening questionnaire and interview process prior to the official data collection period. IS is a male registered chiropractor, and Master of Research student with no prior experience in qualitative research. He received mentoring on qualitative research methodology from HJ and BB. The interview questions (Additional file 1) were developed by the research team to specifically explore the study aims with consideration of previous qualitative studies within the field [20, 30]. Each question included a selection of probing questions that could be used if the interviewer believed that a participant’s response required further exploration or elaboration. IS personally knew 2 study participants as they were colleagues in the Department of Chiropractic, Macquarie University. No prior discussion of the research questions or topic was had with these participants prior to the interviews. No other participants were personally known to IS prior to the commencement of the interviews.

The interviewer did not explicitly state his personal opinion about the research question with participants. Participants were offered the opportunity to review their interview transcripts if they wished. No participants dropped out or refused to complete the interview. No other individuals were present during the interviews. No repeat interviews were performed. All participants received a $50 digital gift card as compensation for their time upon completion of the interview.

### Data analysis

Interview data were analysed in NVivo (Version 12) using framework analysis [31], through an interpretivist lens [32]. This involved a five-step process: (1) familiarisation with the data; (2) indexing (assigning codes to sections of the data); (3) gathering similar codes into preliminary themes; (4) charting data into a thematic framework; and (5) synthesis and interpretation. Two transcripts were initially indexed by IS, HJ and BB individually then compared and discussed to develop an initial framework. Once in agreement, IS indexed the remaining transcripts. This process was performed concurrently with participant recruitment. There were no codes/themes that were generated *a priori*.

### Trustworthiness

Methodological rigour and trustworthiness of the data was addressed throughout the study process [33]. Coding and subsequent development of themes was an iterative and reflective process performed initially by IS and discussed with HJ and BB during regular meetings. Reflexivity was addressed by openly discussing our own perceptions, experiences and biases and how these may influence the development of themes throughout data collection and analysis. To provide dependability and confirmability of the data, IS created dated, handwritten memos for specific codes/themes during interpretation to create an audit trail of the meaning that was derived at that time. This allowed for future critical review when further analysis had been completed and new perspectives had emerged based on exposure to the data. Transferability of our data was addressed by ensuring we produced a thick description of the participants responses, within their individual contexts by highlighting relevant clinical and demographic characteristics.

We determined that data saturation was reached at 17 interviews. At this point the research team were satisfied there was a sufficient breadth and depth of the data and further interviews were not identifying new data.

## Results

### Participant characteristics

Seventeen chiropractors were interviewed. Participant characteristics are outlined in Table 1. Interview duration ranged from 11 to 49 min, with an average of 18 min. Most participants were male (*n* = 11/17, 64%) and the majority practiced in New South Wales (*n* = 13/17, 76%). Years in practice ranged from one to 38, with nine participants practicing for greater than 20 years (9/17, 53%).

**Table 1:** Participant characteristics

Characteristic	N (%)
*Sex*	
Male Female	11 (65) 6 (35)
*State/Territory of practice*	
New South Wales Queensland Victoria South Australia Australian Capital Territory	13 (76) 1 (6) 1 (6) 1 (6) 1 (6)
*Location of practice*	
Urban Rural	15 (88) 2 (12)
*Years in practice*	
<10 11–20 >20	3 (18) 5 (29) 9 (53)
*Institution of study*	
Macquarie University Central Queensland University Royal Melbourne Institute of Technology Life University Durban Institute of Technology Palmer Davenport	11(65) 1 (6) 2 (12) 1 (6) 1 (6) 1 (6)
Techniques used in practice*	
Diversified Gonstead Instrument adjusting Drop-piece adjusting Flexion distraction Activator methods Thompson technique Sacro-occipital technique (SOT) Chiropractic BioPhysics (CBP) Scoliosis specific rehabilitation Atlas Orthogonal	13 (76) 2 (12) 11 (65) 12 (71) 3 (18) 4 (24) 3 (18) 4 (24) 5 (29) 2 (12) 1 (6)
Reasons likely to refer for radiography*	
Clinical suspicion of serious pathology (e.g., cancer, infection, inflammatory arthritis) Clinical suspicion of fracture Screening for contraindications for spinal manipulation, without clinical suspicion Clinical suspicion of benign spinal pathology (e.g., degeneration, spondylolisthesis) Biomechanical analysis (e.g., spinal listings, spinal curve assessment) At a patient’s request or for patient reassurance	15 (88) 12 (71) 4 (24) 13 (76) 11 (65) 4 (24)
*Proportion of patients typically referred for radiography*	
<10% 10–20% 21–50% 51–80% >80%	2 (12) 3 (18) 4 (24) 3 (18) 5 (29)
*Location radiographs obtained*	
In-house facilities Medical radiology practice	5 (29) 12 (71)

*Participants could choose multiples responses

### Themes

Descriptive themes were developed with respect to each aim of the study. Aim 1 explored how chiropractors determine when to order radiographs, for which three themes were developed. Aim 2 explored how chiropractors use radiographs to inform their clinical management, for which three themes and four subthemes were developed.

### Aim 1: How chiropractors determine when to order radiographs

**Fig. 1:**
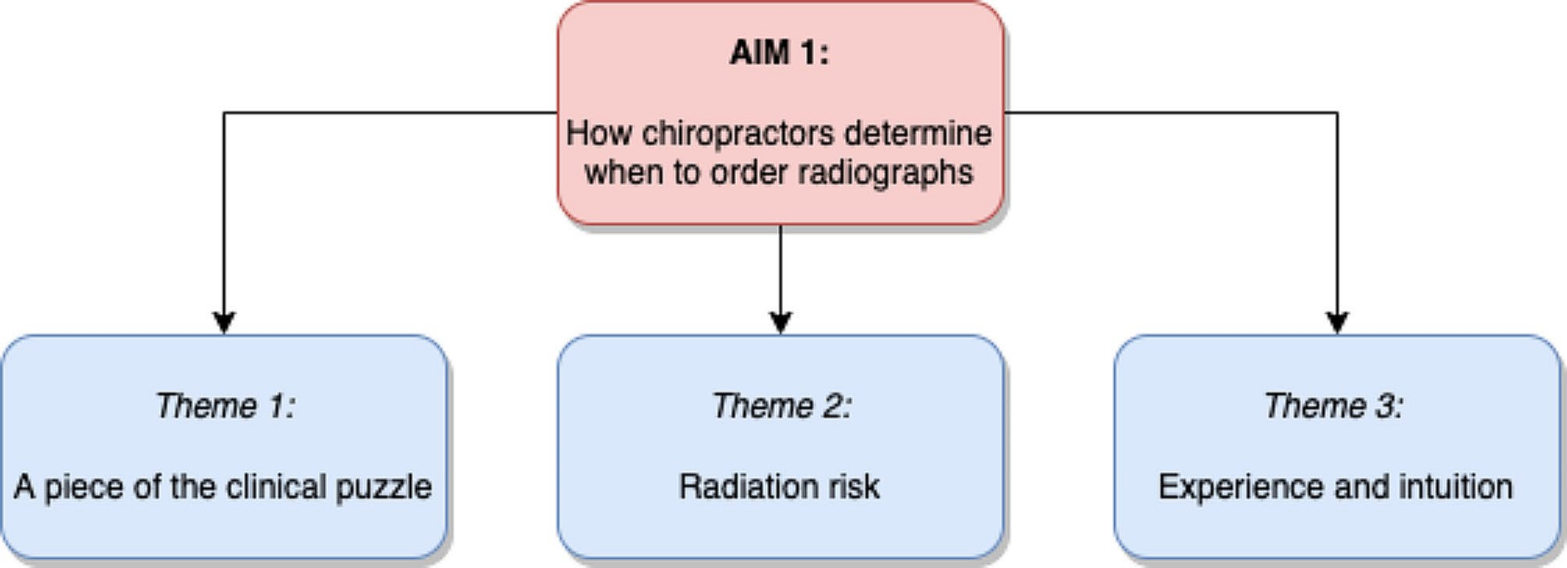
Aim 1 themes

Chiropractors’ decisions of when to refer for spinal radiographs were influenced by the perceived impact that imaging findings might have on clinical management, the chiropractors’ perception of radiation risk, and the chiropractors’ clinical experience. Figure 1 illustrates the three themes that were developed.

### Theme 1: A piece of the clinical puzzle

The overarching question that participants appeared to ask themselves to inform their decision to order radiographs was - “Will information from the images change my management of this patient?”. However, the degree to which radiographic findings were perceived to change management existed on a spectrum. Most participants emphasised the importance of identifying potentially serious pathology that would require referral for medical care, explaining the role of appropriate history taking, differential diagnosis, and physical examination to determine the necessity for radiographs:

*“If I’ve got a 65-year-old who’s decided to jump on the trampoline with the grandkids and felt something go in her thoracic spine, and with instant pain… I’m taking an X-ray and I’m looking for a compression fracture and I’m not touching it” (Participant 11 (P11), Male (M), 30 years in practice, 21–50% radiography referral).*

Participants were also cognizant of the role of trialling conservative care prior to ordering a radiograph, and the ability to deviate from the initial care plan when necessary. The duration of a trial of care differed between participants, from a few visits to four to six weeks. Many reported they would do a trial of care as recommended by CPGs, and only order imaging if the patient did not respond favourably:

*“… my clinical practice is more so guided by guidelines I would say. So, things that align to that… either someone who’s not responded to treatment after a trial of care for about 6 weeks or gotten worse obviously” (P12, Female (F), 4 years in practice, < 10% radiography referral).*

Other participants described their rationale for using radiographs in the assessment of more benign spinal presentations, where specific information from the radiograph would potentially direct the most appropriate treatment for the patient:

*“… if you think the spine is imbalanced… then the only way to determine the most appropriate treatment protocol is to take an X-ray. Because you know, just in the simple case of forward head posture, you know, forward head posture can be driven from the thoracic spine, or it can be primarily a neck problem… so measuring the thoracic spine is essential in determining how to address a forward head posture” (P10, M, 17 years in practice, > 80% radiography referral).*

This quote also demonstrates the perception that some participants held around a need to visualise the structure of the spine and extremities, which appeared to drive their rationale for ordering radiographs. Participants referred to the importance of assessing and measuring structural abnormalities, with the view that certain abnormalities such as scoliosis could increase the risk of harm or adverse events and could alter management decisions:

*“… it’s impossible to understand the intricacies of the condition [scoliosis], the severity, without an X-ray… also your treatment protocol which can be a life changing treatment protocol, is based on specifics that are [only] seen on the X-ray” (P8, M, 22 years in practice, 21–50% radiography referral).*

### Theme 2: Radiation risk

Conflicting perceptions were described by participants regarding the potential risk of ionising radiation from spinal radiographs. Some participants perceived that the benefits of spinal radiographs would generally outweigh the potential risk from radiation exposure, citing small dosages of radiation that would be unlikely to create harm:

*“I think it [radiography] definitely improves health outcomes, especially considering how small the dosage is… I think it’s very justified” (P6, M, 11 years in practice, 21–50% radiography referral).*

Whereas other participants described their concern about potential radiation risk and would trial conservative care to determine how a patient would respond, before ordering a radiograph:

*“… if I’m erring on the side of caution and that is the caution not to expose them to radiation, you can go a couple of visits… if there’s improvement in range of motion without an X-ray, you don’t need it” (P17, M, 25 years in practice, 51–80% radiography referral).*

### Theme 3: Experience and intuition

Participants alluded to previous clinical experience, both personal and inter-personal, as potentially influencing their decision to order spinal radiographs. For example, if obtaining a radiograph had appeared to inform care or provide value in the past, the chiropractor would be more likely to adopt this strategy in the future:

*“I think there is too often times where I will receive an X-ray back and think I’m really glad I’ve got an X-ray of this patient. Which then drives me to… err on the side of anything that concerns me or anything, that I’m sort of getting an X-ray” (P9, F, 26 years in practice, > 80% radiography referral).*

In contrast, another participant shared their perspective, where radiography did not seem to play such a pivotal role in their management, despite having many years of clinical experience:

*I’ve never given a treatment then afterwards thought damn, if only I had that X-ray first” (P14, F, 19 years in practice, < 10% radiography referral).*

More clinical experience also helped some participants be more certain in their clinical decision making and deciding when to refer for radiographs. One participant reflected on how their utilisation of radiography has decreased over time, compared to when they were a new graduate, as they feel more confident in their clinical skills for determining when radiographs will impact management:

*“I feel much more settled as to when to choose to get an X-ray and not. I find [that’s] just that clinical experience. So, when I was a newer grad, I think I wanted to X-ray everybody because I felt like I didn’t know… you’re just kind of grasping, aren’t you?” (P16, F, 30 years in practice, 10–20% radiography referral).*

Participants also referred to the role of intuition, or a “gut feel” to guide their decisions as to when radiographs would be appropriate, even when specific clinical indications were not apparent:

*“… it’s just you’ve been around long enough to smell something, you know? You’ve been around long enough because it’s not a magical thing. It just… seems intuitive. Because it’s embedded into your psyche through experience and you’ll go, you know, I think I will X-ray this person, you know, and then you find something! So yeah, I think what that is [is] just collective wisdom and knowledge” (P17, M, 25 years in practice, 51–80% radiography referral).*

Although clinical intuition may be heavily influenced by previous experience, this does not seem to be the only factor that influences the use of intuition in the clinical decision-making process. For example, one chiropractor, with only 1-year clinical experience, described using intuition when discussing uncertainty around what physical exam findings may indicate the need for radiographs:

*“That’s something that is a gut feel” (P1, F, 1 year in practice, 10–20% radiography referral).*

### Aim 2: How radiographs inform chiropractors’ clinical management

**Fig. 2:**
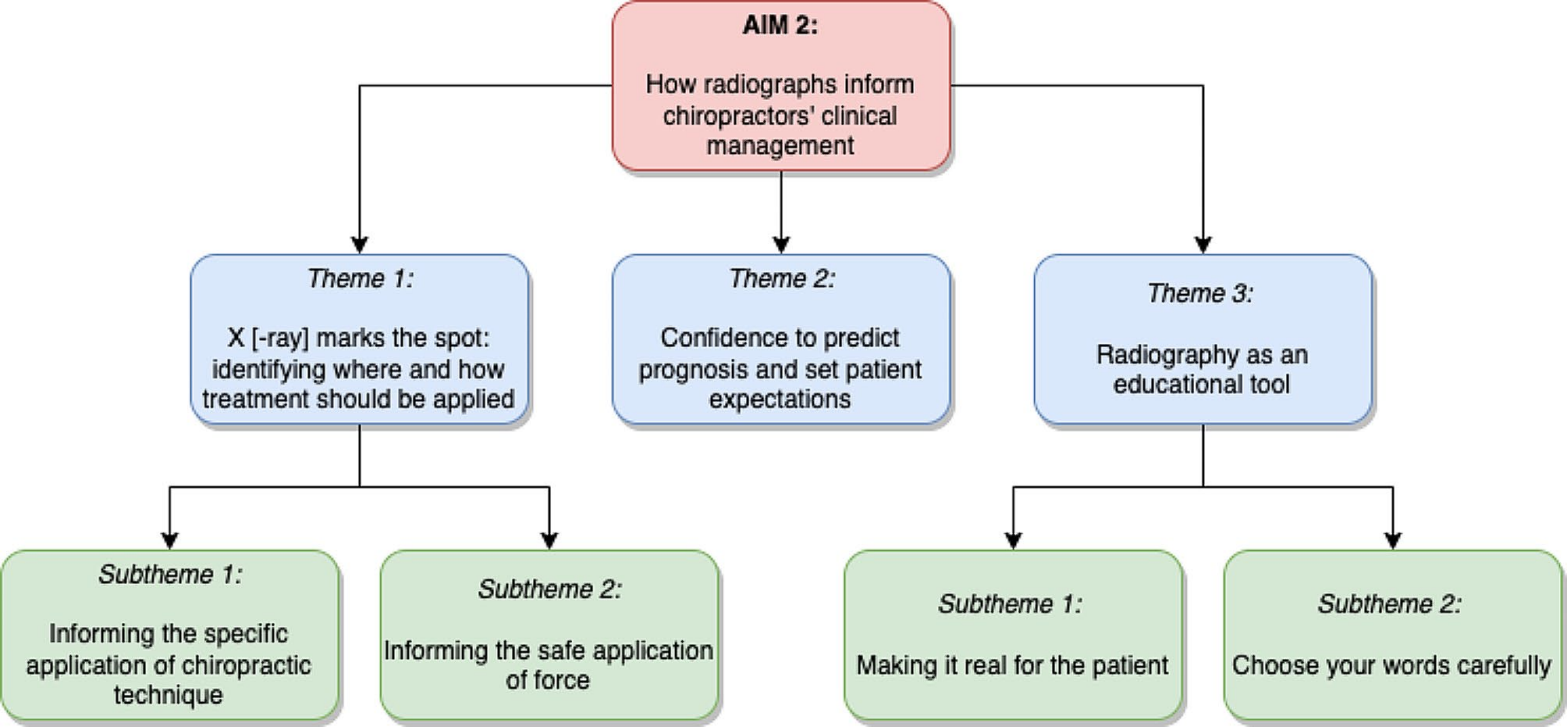
Aim 2 themes and subthemes

The first theme describes alterations, or lack thereof, in their specific application of a technique or a treatment approach. The second theme relates to when obtaining radiographs gives chiropractors more confidence in their management plan by informing patient prognosis and setting expectations. The third theme describes using radiographs as an educational tool, which is used to convey meaning to patients and explain findings in a way that is perceived to reassure the patient. Figure 2 illustrates the three themes and four subthemes.

### Theme 1: X [-ray] marks the spot: identifying where and how treatment should be applied

Just as chiropractors’ rationale for ordering radiographs exists on a spectrum, so too does their use of radiographs to guide the application of chiropractic technique. Some participants appeared to extract substantial detail from radiographs which they used to inform treatment application, whereas others felt that radiographs did not inform their application of treatment at all. For some, radiographic findings changed where treatment was to be applied (spinal region), or what type of treatment was to be given (e.g. exercise or the use of an orthosis). However, most participants referred to using radiographs to determine the appropriateness of applying physical forces to the spine, and to identify contraindications or indications for referral. Two subthemes were developed.

### Subtheme 1: Informing the specific application of chiropractic technique

Some participants reported assessing spinal radiographs to determine how to apply a specific technique, which they perceived to enhance clinical outcomes. This included reference to ‘named’ technique systems that are used by some chiropractors. Some technique systems use a pre-determined protocol for the chiropractor to follow, which may use information from radiographs to guide treatment, specifically the application of SM, or a ‘chiropractic adjustment’:

*“… in the case of Atlas Orthogonal, it [radiography] does tell you what angle and which direction, so it provides a vectored adjustment which, we pretty much put up there as a hallmark of why we get results with so little force. Is that because we only use a very tiny 14 newtons to adjust, hand adjustments are about 400 [newtons]… We believe that’s because of the specificity, offered by the X-ray analysis” (P17, M, 25 years in practice, 51–80% radiography referral).*

Participants who specifically manage patients with abnormal spinal curvatures emphasised the importance of radiographic information to guide the application of treatment. This included the need for specific details to build orthoses for patients with scoliosis:

*“The accuracy of our implementation of treatment modality is based on the X-ray… when a device is being built to help control this scoliosis, you need accurate information. You can’t build on postural features, so it’s [radiography] absolutely 100% important” (P8, M, 22 years in practice, 21–50% radiography referral).*

As well as how and where to apply a manual traction technique designed to restore normal spinal curvature such as cervical lordosis:

*“… you’ll change the forces that you use in the traction, the position that you use where you put the harnesses, all that based on what you see on the X-ray” (P3, M, 21 years in practice, > 80% radiography referral).*

In contrast, other participants did not consider that the use of radiographs was necessary to inform the application of chiropractic techniques. These participants placed little value on assessing for or correcting the specific orientation of individual spinal segments:

*“… but not really in the exact manner of oh, because I can see that their L3 is rotated by two degrees, I’m going to be trying to put two degrees of rotation. I’m not into that at all, and I don’t think it makes a difference personally” (P6, M, 11 years in practice, 21–50% radiography referral).*

### Subtheme 2: Informing the safe application of force

Identifying specific spinal abnormalities on radiographs was considered very important by some participants. These participants felt that specific radiographic findings helped them modulate the level of force applied during chiropractic techniques. Findings such as osteoporosis, spondylolisthesis, degeneration, or transitional segments were stated as examples where a practitioner might modify how or where they applied manual forces:

*“… if someone was quite osteoporotic, I’d be a lot more gentle and careful, and acknowledge that they [the patient] were a potential risk” (P6, M, 11 years in practice, 21–50% radiography referral).*

Participants also described how radiographic findings changed the region of the spine that they were treating or intending to treat. This was often accompanied by a perceived inconsistency between the radiographic findings and their physical exam findings:

*“… a lot of the time like there’s only so much like I can find like with when I assess… I had a patient the other day, [she] gets migraines, has for her whole life. Tenderness, palpation, everything showed C7. On the X-ray… I found that [it] was a C4 issue. Didn’t pick it up with the palpation” (P15, M, 1 year in practice, > 80% radiography referral).*

### Theme 2: Confidence to predict prognosis and set patient expectations

Many participants described how structural findings or biomechanical abnormalities on radiographs can be useful in predicting the patients’ prognosis. This was commonly in reference to benign findings such as degeneration:

*“… if I’ve got some level of osteoarthritis or degeneration there [on the radiograph], it will indicate to me that it’s going to be a slower healing period” (P14, F, 19 years in practice, < 10% radiography referral).*

Some participants also referred to the presence of abnormal spinal curvatures as having predictive value for symptom recurrence. These participants stated that a patient’s risk could be reduced by changing the patient’s curves through treatment:

*“… if someone has a reduced lumbar curve, hypolordosis, the probability is, that that’s not normal based on the morphology. I know the probability of them having reoccurring problems in the future is higher than if I can restore the spinal curves towards normal or to normal” (P7, M, 38 years in practice, 51–80% radiography referral).*

Viewing their patient’s prognosis through a structural/biomechanical lens based on radiographic findings, also allowed for participants to decrease potentially unrealistic patient goals or expectations. One participant described how he used radiographic findings to show a patient with post-Scheuermann’s disease that there were structural changes that were responsible for their increased thoracic kyphosis, and that the idea of changing their curve was unrealistic:

*“… I can also take some pressure off myself that the patient’s putting on me to make their spine straight, and then they’re going to be blaming me when they’re spine isn’t straight… I suppose if I’m really honest, there might be some part in me that [will use the radiograph] because I want to give the patient a realistic view of their prognosis” (P2, M, 23 years in practice, 21–50% radiography referral).*

### Theme 3: Radiography as an educational tool

Theme 3 explores how participants used radiographs during their clinical encounters with patients to provide education in a way that they believe would give confidence and reassurance to the patient. Two subthemes were developed. Subtheme 1 describes how radiographic findings are used to explain the diagnosis or symptoms to patients in a way that resonates with them. Subtheme 2 describes the important role of communication, and how explaining radiographic findings is perceived as being reassuring for some patients.

### Subtheme 1: Making it real for the patient

Radiographic findings are not inherently meaningful until they are interpreted and communicated to the patient. Providing education about the patient’s spine using radiographs, explaining their symptoms, and contextualising their experience, was considered important for many participants. For some, radiography was part of the bigger clinical picture, where they used radiographic findings to help give patients a better understanding of their condition:

*“… any information that the patient presents with, if they present with a bunch of scans and X-rays or I’ve ordered something … After I’ve heard their story, I try to synthesize it all back together and tell their story back to them in a way that helps them understand what’s going on with them better” (P2, M, 23 years in practice, 21–50% radiography referral).*

*“… and you might always have to bring them back to it, you say, ‘remember why you’re having the tingling in the hand? Let me show you that disk’. So, you can settle them down pretty quickly when they’ve got spinal degeneration or a reason that you can point to [on] the X-ray” (P17, M, 25 years in practice, 51–80% radiography referral).*

Other participants described how having a diagnosis or being able to show a structural finding on radiographs can help make the condition *real* for the patient. They perceived that the condition was something tangible, that the patient could see and that this could help improve patient compliance with treatment:

*“… if you point that out that, you know your poor old neck is in such a horrendous forward head carriage, and no one’s ever pointed that out, that structural effect. Yeah, you’ll get big ‘aha’ moments where the patient will go: ‘well, I’m glad I came to you’” (P17, M, 25 years in practice, 51–80% radiography referral).*

*“… then it’s [the radiograph] helping with that compliance from an aspect of saying, hey, this is this is what you’re feeling when you tell me that your postures like this, this is what’s going on the under the surface and this is the reason why these things [are] going to be necessary for you to do at home” (P5, M, 12 years in practice, > 80% radiography referral).*

#### Subtheme 2: Choose your words carefully

Many chiropractors were cognizant of the potential negative effects or connotations that certain radiographic findings may have on patients. Moreover, participants believed that these negative effects could be minimised by communicating with patients in a way that doesn’t create excessive fear or worry about their condition. Some chiropractors described the reassuring value that reporting radiographic findings, or lack thereof, can have on patients. Especially those that are anxious about the potential cause of their pain:

*“… some patients are very anxious anyway, and often they’re the ones that actually will want to be X-rayed because they’re anxious that there’s something else going on there. When they see an X-ray and there’s nothing on it, suddenly miraculously, they’re 50% better… The healing X-ray” (P16, F, 30 years in practice, 10–20% radiography referral).*

One participant reflected on how they communicate radiographic findings to their patients, and described intentionally modifying their communication due to concern for the potential harm it could create:

*“… I think I’m very aware of speaking to patients in ways that doesn’t create another injury to their mind, in the way that I report findings on their X-rays” (P2, M, 23 years in practice, 21–50% radiography referral).*

The same participant also explained how different patients may respond differently to the reporting of an absence of significant radiographic findings, specifically for people with chronic spinal pain:

*“… for them [patients with chronic spinal pain] seeing normal X-rays can go either way… it can reinforce the idea that they’re mad and that nobody believes in them because they’ve got normal X-rays. And so, they’re very suspicious that I’m [the chiropractor] going to be thinking, yep, you are a nutter. You’ve just got imaginary pain or something. Or it can be like reassuring to them” (P2, M, 23 years in practice, 21–50% radiography referral).*

## Discussion

### Comparison to other literature

This study, as the first to explore the perceptions of Australian chiropractors on the use of radiography in chiropractic practice, had several important novel findings, as well as sharing similarities with previous research. Consistent with previous qualitative research [25, 26], this study identified the potential influence of external factors, such as prior clinical experience, and how these may impact a chiropractor’s decision to order spinal radiographs. In particular, clinical experience appeared to be inherently intertwined with future clinical decisions. In the context of this study, prior experience may influence a chiropractor’s ability to accurately consider risk, if, their perception of risk has been skewed by previous experience. For example, making a serendipitous discovery on imaging of an epidemiologically rare and serious condition may lead to a higher perception of risk of missing such conditions if imaging is not ordered in the future.

Although the reasoning for ordering radiographs, and how they were used to inform management varied among participants in this study, overall, most reported that they only use radiography in situations where they believed it would be of benefit to the patient. Many emphasised their adherence to evidence-based CPGs, which has been highlighted in previous qualitative research [26]. However, some of the potential benefits or ways of using radiographs discussed by participants are not well supported by current evidence [9, 17]. An important finding in this study is the role that chiropractors perceived radiographs to have in determining patient prognosis and using that to set patient expectations about the likelihood of recovery and recurrence of symptoms. Prognostic recommendations described were often based on the presence of degenerative findings or abnormal spinal curvatures, which may be inconsistent with current evidence, given the unclear relationship between diagnostic imaging findings and future lower back [14] and neck pain [15] Prognosis has been reported as a reason for ordering diagnostic imaging by chiropractors in two previous surveys. De Zoete et al. [35] and Assendelft et al. [34] found 54% and 92% of respondents reported diagnostic imaging is ‘often or always’ indicated for prognosis, respectively. However, no detail about how prognosis is determined was provided in these surveys.

Perceptions on how participants use radiographs to inform the application of chiropractic technique existed on a spectrum in our study. Bussières et al [25]. demonstrated that some chiropractors believe that a proposed treatment should change in scenarios where benign spinal abnormalities (e.g., transitional segments) are found on imaging. However, what exactly the chiropractor changes about their treatment was not explored. Participants in our study shared in significant detail how they used minute changes in force and vectors when using manual therapy techniques such as SM, as well as where specifically they choose to apply these techniques. Conversely, many participants in our study, as in previous studies [25, 26] did not consider imaging important to inform how they applied chiropractic technique, beyond the presence of serious pathology.

Some participants in this study perceived value in using spinal radiographs for patient education. They felt that providing a specific structural diagnosis or finding on the radiograph helped to make the condition real for the patient and provide reassurance that a serious cause is not evident. This finding is consistent with previous literature exploring clinicians’ beliefs about diagnostic imaging for LBP in other professions [36]. However, as one participant in the current study reflected upon, the patient may not share this belief, and the way radiographic findings are reported to patients may have potentially negative effects. This belief is shared by Italian osteopaths who emphasised the potential nocebic impact spinal imaging can have on LBP patients [37].

Varied responses to imaging have been highlighted by Carlin et al. [38] who explored primary care patients’ perceptions of viewing their own diagnostic images, with some participants reporting increased anxiety as opposed to reassurance when viewing imaging findings. Terms that are commonly provided in diagnostic imaging reports such as ‘disc degeneration’ are poorly understood by the general population [39], may increase patients perceived need for imaging [40], and may not, therefore, be inherently reassuring. Strategies have been investigated to try and reduce unintended harm associated with the reporting of radiographic findings [41].

### Strengths and limitations

Multiple sampling strategies, including purposive sampling were used with the intent of capturing a wide variety of chiropractors’ views and perceptions. The majority of included participants were male and practicing in urban locations, which is consistent with Australian national workforce statistics [42]. No significant response variation was noted between participants with these different characteristics. Sufficient breadth of participant characteristics likely to impact the research question (e.g., clinical experience, imaging use, institution of study) were captured, ensuring transferability of our results [43]. Having a prior collegial relationship with two participants may have introduced potential bias, however this was mitigated by not discussing the research questions or topic prior to the interviews. A methodological limitation of this study was not seeking revision of transcripts or results from interview participants (member checking). This practice aims to verify that the results adequately reflect the meaning the participant intended. Additionally, we did not perform peer debriefing with researchers outside of our team. Addressing these aspects of trustworthiness would have strengthened the credibility of our results [44].

An area that was not addressed in the questionnaire or during interviews was reimbursement for radiographic services, or the influence of having in-house X-ray facilities. It is possible that financial incentives are a potential driver for ordering spinal radiographs for some chiropractors [25].

This qualitative research study was designed to explore the breadth of opinions and perceptions of the participating chiropractors, rather than produce accurate estimates regarding sample or population characteristics of Australian chiropractors. It is therefore important to note that the participant opinions in this study do not necessarily reflect those of all chiropractors, including those who share similar characteristics. For example, there are a variety of techniques used by chiropractors, and not all technique systems used by chiropractors have been represented in this study. Furthermore, in scenarios where a technique was mentioned explicitly, it cannot be assumed that the practitioner using that particular chiropractic technique was adhering faithfully to the protocols set out by the technique creators.

### Implications and future research

This study highlights and explores the variation in Australian chiropractors’ perceptions regarding the use of spinal radiography in clinical practice and is the first study to explore how radiographs inform the clinical management of patients with spinal disorders. Given the implementation of public health initiatives to encourage the appropriate use of diagnostic imaging, such as choosing wisely [45], and specific commentary within the chiropractic profession [9], it is important to understand what influences and drives individuals to make clinical decisions, especially for situations that are not currently supported by research evidence. Specifically, findings from this study relating to the use of radiography to inform the application of chiropractic technique, it’s role in predicting prognosis, and its use as an educational tool are areas that warrant further research. It is unclear to what extent these uses of spinal radiography affect patients with spinal disorders. Participants in this study emphasised the importance of how radiography findings are reported to patients, therefore future research should explore what information is provided, as well as how it is delivered. By improving what information is provided to patients about their imaging finding, and how it is reported, potential harms may be reduced or avoided, and clinical benefits may be enhanced.

## Conclusion

Australian chiropractors’ decision-making around spinal radiography is diverse and can be influenced by a number of personal, clinical, and other external factors. Reasoning for ordering radiographs revolved around the chiropractor’s perception of how their clinical management might be influenced by radiographic findings. The frequency or intention to order radiographs was influenced by their perception of radiation risk, as well as clinical experience and intuition. Radiography was perceived to inform the application of chiropractic treatment to varying degrees. Chiropractors perceived radiography to be useful for other aspects of clinical management beyond technique selection and application, such as predicting prognosis, and as an educational tool. Previously unexplored uses of spinal radiography in clinical practice were highlighted. Some chiropractors reported benefits of radiography that are currently not supported by research evidence. Future research should address how radiographic findings are reported to patients with spinal disorders and how this could be optimised to improve patient outcomes.

### Electronic supplementary material

Below is the link to the electronic supplementary material.

Supplementary Material 1

Supplementary Material 2

Supplementary Material 3

## Data Availability

The data collected in this study may be made available to researchers, in a non-identifiable form, for future Human Research Ethics Committee approved research projects which are an extension of, or closely related to, this proposed study. Unique identifying numbers will be removed, and random numbers will be used to code the data, which cannot be later re-identified.

## Abbreviations

CPGs: Clinical practice guidelines
LBP: Low back pain
SM: Spinal manipulation
US: United states

## References

[1] Kamper SJ, Logan G, Copsey B, Thompson J, Machado GC, Abdel-Shaheed C (2020). What is usual care for low back pain? A systematic review of health care provided to patients with low back pain in family practice and emergency departments. Pain.

[2] Downie A, Hancock M, Jenkins H, Buchbinder R, Harris I, Underwood M (2020). How common is imaging for low back pain in primary and emergency care? Systematic review and meta-analysis of over 4 million imaging requests across 21 years. Br J Sports Med.

[3] Logan GS, Pike A, Copsey B, Parfrey P, Etchegary H, Hall A (2019). What do we really know about the appropriateness of radiation emitting imaging for low back pain in primary and emergency care? A systematic review and meta-analysis of medical record reviews. PLoS ONE.

[4] Jenkins HJ, Downie AS, Maher CG, Moloney NA, Magnussen JS, Hancock MJ (2018). Imaging for low back pain: is clinical use consistent with guidelines? A systematic review and meta-analysis. Spine J.

[5] McDonald MA, Kirsch CFE, Amin BY, Aulino JM, Bell AM, Cassidy RC (2019). ACR appropriateness Criteria^®^ cervical neck pain or cervical radiculopathy. J Am Coll Radiol.

[6] Jones JY, Saigal G, Palasis S, Booth TN, Hayes LL, Iyer RS (2019). ACR appropriateness criteria^®^ scoliosis-child. J Am Coll Radiol.

[7] Hutchins TA, Peckham M, Shah LM, Parsons MS, Agarwal V, Boulter DJ (2021). ACR appropriateness criteria^®^ low back pain: 2021 update. J Am Coll Radiol.

[8] Lemmers GPG, van Lankveld W, Westert GP, Van der Wees PJ, Staal JB (2019). Imaging versus no imaging for low back pain: a systematic review, measuring costs, healthcare utilization and absence from work. Eur Spine J.

[9] Jenkins HJ, Downie AS, Moore CS, French SD (2018). Current evidence for spinal X-ray use in the chiropractic profession: a narrative review. Chiropr Man Th.

[10] Medicare item reports July 2019 - June 2020. Accessed 13. May 2024. http://medicarestatistics.humanservices.gov.au/statistics/mbs_item.jsp. 2020.

[11] Webster BS, Choi Y, Bauer AZ, Cifuentes M, Pransky G (2014). The cascade of medical services and associated longitudinal costs due to nonadherent magnetic resonance imaging for low back pain. Spine (Phila Pa 1976).

[12] Jacobs JC, Jarvik JG, Chou R, Boothroyd D, Lo J, Nevedal A (2020). Observational study of the downstream consequences of inappropriate MRI of the lumbar spine. J Gen Intern Med.

[13] Brinjikji W, Luetmer PH, Comstock B, Bresnahan BW, Chen LE, Deyo RA (2015). Systematic literature review of imaging features of spinal degeneration in asymptomatic populations. Am J Neuroradiol.

[14] Steffens D, Hancock MJ, Maher CG, Williams C, Jensen TS, Latimer J (2014). Does magnetic resonance imaging predict future low back pain? A systematic review. Eur J Pain.

[15] Hill L, Aboud D, Elliott J, Magnussen J, Sterling M, Steffens D (2018). Do findings identified on magnetic resonance imaging predict future neck pain? A systematic review. Spine J.

[16] Stochkendahl MJ, Rezai M, Torres P, Sutton D, Tuchin P, Brown R (2019). The chiropractic workforce: a global review. Chiropr Man Th.

[17] Bussières A, Peterson C, Taylor JAM (2008). Diagnostic imaging practice guidelines for musculoskeletal complaints in adults — an evidence-based approach — part 3: spinal disorders [practice guideline]. J Manipulative Physiol Ther.

[18] Harrison D, Harrison D, Kent C, Betz J (2009). Practicing chiropractors’ Committee on Radiology Protocols (PCCRP) for Biomechanical Assessment of spinal subluxation in Chiropractic Clinical Practice.

[19] Arnone PA, Kraus SJ, Farmen D, Lightstone DF, Jaeger J, Theodossis C (2023). Examining clinical opinion and experience regarding utilization of plain radiography of the spine: evidence from surveying the Chiropractic Profession. J Clin Med.

[20] Jenkins HJ. Awareness of radiographic guidelines for low back pain: a survey of Australian chiropractors. Chiropr Man Th. 2016;24(39). 10.1186/s12998-016-0118-7 PMC5051064 27713818

[21] Oakley PA, Harrison DE. Radiogenic cancer risks from chiropractic x-rays are zero: 10 reasons to take routine radiographs in clinical practice. Ann Vert Sublux Res. 2018;3(10).

[22] Lopes MA, Coleman RR, Cremata EJ (2021). Radiography and clinical decision-making in Chiropractic. Dose-Response.

[23] Jenkins HJ, Kongsted A, French SD, Jensen TS, Doktor K, Hartvigsen J (2021). What are the effects of diagnostic imaging on clinical outcomes in patients with low back pain presenting for chiropractic care: a matched observational study. Chiropr Man Th.

[24] Corso M, Cancelliere C, Mior S, Kumar V, Smith A, Cote P (2020). The clinical utility of routine spinal radiographs by chiropractors: a rapid review of the literature. Chiropr Man Th.

[25] Bussières AE, Patey AM, Francis JJ, Sales AE, Grimshaw JM, Brouwers M (2012). Identifying factors likely to influence compliance with diagnostic imaging guideline recommendations for spine disorders among chiropractors in North America: a focus group study using the theoretical domains Framework. Implement Sci.

[26] To D, Hall A, Bussières A, French SD, Lawrence R, Pike A (2022). Exploring factors influencing chiropractors’ adherence to radiographic guidelines for low back pain using the theoretical domains Framework. Chiropr Man Th.

[27] Ammendolia C, Bombardier C, Hogg-Johnson S, Glazier R (2002). Views on radiography use for patients with acute low back pain among chiropractors in an Ontario community. J Manipulative Physiol Ther.

[28] Tong A, Sainsbury P, Craig J (2007). Consolidated criteria for reporting qualitative research (COREQ): a 32-item checklist for interviews and focus groups. Int J Q Health Care.

[29] Moser A, Korstjens I, Series (2018). Practical guidance to qualitative research. Part 3: Sampling, data collection and analysis. Eur J Gen Pract.

[30] De Carvalho D, Bussières A, French SD, Wade D, Brake-Patten D, O’Keefe L et al. Knowledge of and adherence to radiographic guidelines for low back pain: a survey of chiropractors in Newfoundland and Labrador, Canada. Chiropr Man Th. 2021;29(1). 10.1186/s12998-020-00361-2 PMC7812732 33461555

[31] Ritchie J, Spencer L, O’Connor W (2003). Carrying out qualitative analysis. Qualitative Res Practice: Guide Social Sci Students Researchers.

[32] Willis J. Foundations of qualitative research: interpretive and critical approaches. Sage; 2007.

[33] Nowell LS, Norris JM, White DE, Moules NJ. Thematic analysis: striving to meet the trustworthiness Criteria. Int J Qual Methods. 2017;16(1).

[34] Assendelft WJJ, Pfeifle CE, Bouter LM (1995). Chiropractic in the Netherlands: a survey of Dutch chiropractors. J Manipulative Physiol Ther.

[35] de Zoete A, de Boer MR, van Tulder MW, Rubinstein SM, Ostelo R (2022). Diagnostic imaging in Chiropractic Practice: a survey of opinions and self-reported Guideline Adherence of Dutch and Belgian chiropractors. J Manipulative Physiol Ther.

[36] Sharma S, Traeger AC, Reed B, Hamilton M, O’Connor DA, Hoffmann TC (2020). Clinician and patient beliefs about diagnostic imaging for low back pain: a systematic qualitative evidence synthesis. BMJ Open.

[37] Ogliari L, Formica A, Bettelli L. ‘More harm than good’ – a qualitative study exploring the attitudes and beliefs of a group of Italian osteopaths about spinal imaging in the management of patients with chronic low back pain. Int J Osteopath Med. 2023;50.

[38] Carlin LE, Smith HE, Henwood F (2014). To see or not to see: a qualitative interview study of patients’ views on their own diagnostic images. BMJ Open.

[39] Farmer C, O’Connor DA, Lee H, McCaffery K, Maher C, Newell D (2021). Consumer understanding of terms used in imaging reports requested for low back pain: a cross-sectional survey. BMJ Open.

[40] O’Keeffe M, Ferreira GE, Harris IA, Darlow B, Buchbinder R, Traeger AC (2022). Effect of diagnostic labelling on management intentions for non-specific low back pain: a randomized scenario‐based experiment. Eur J Pain.

[41] Witherow JL, Jenkins HJ, Elliott JM, Ip GH, Maher CG, Magnussen JS (2022). Characteristics and effectiveness of interventions that Target the Reporting, Communication, or clinical interpretation of lumbar imaging findings: a systematic review. Am J Neuroradiol.

[42] National Health Workforce Dataset. Chiropractors 2015–2019. Accessed May 14 2024. https://hwd.health.gov.au.

[43] Korstjens I, Moser A, Series. Practical guidance to qualitative research. Part 4: trustworthiness and publishing. Eur J Gen Pract. 2018;24(1). 10.1080/13814788.2017.1375092 PMC8816392 29202616

[44] Kornbluh M. Combatting challenges to establishing trustworthiness in qualitative research. Qual Res Psychol. 2015;12(4).

[45] Rao VM, Levin DC. The overuse of diagnostic imaging and the choosing wisely initiative. Volume 157. Ann Intern Med; 2012. pp. 574–6. 10.7326/0003-4819-157-8-201210160-00535 22928172

