# Supplementary material for: Chiropractors’ perceptions on the use of spinal radiographs in clinical practice: a qualitative study
Source: Chiropr Man Therap. 2024 Jun 22;32:23. doi: 10.1186/s12998-024-00547-y (PMC11193277; doi:10.1186/s12998-024-00547-y)
Supplement: Supplementary file 1 — Supplementary Material 1 [file 12998_2024_547_MOESM1_ESM.docx]

Additional file 1 – Interview questions

**Introduction:**

Thank you for participating in this interview. This study will ask you questions about how you use spinal X-rays in clinical practice as a chiropractor. There are no right or wrong answers; we interested in all types of views about the use of X-rays in chiropractic clinical practice.

As we discuss your ideas and opinions, you can reflect generally on your experiences as a chiropractor and cases that you have managed. Please do not provide any details related to specific patients or cases that would impact patient confidentiality.

Everything you say will be strictly confidential and your name will be removed from the transcripts.

I would like to audio-record the interview so I can listen more carefully instead of writing notes. Audio recordings will be transcribed with any identifying names removed. You will not be identifiable in the interview transcript. You will be provided with the opportunity to review and edit a transcript of this interview if you wish. Do I have your permission to record this interview?

The interview will last about 20-30 minutes. If there are any questions you don’t want to answer just let me know, and you can stop the interview at any time.

*(Start recording)*

**Question 1:** How do you determine which patients need to be referred for spinal X-rays?

Probes:

- How frequently would you normally refer patients for X-rays and why? (initial X-rays and repeat X-rays if used)
- Are there specific clinical practice guidelines that you use to guide your decision making? If so, which and how do you use them?
- What rationale do you use to determine who needs X-rays (e.g. suspicion of pathology/trauma, screen for contraindications, confirm diagnosis, biomechanical assessment, patient pressure, medicolegal pressure, etc.)
- Are there specific clinical findings that indicate the need for X-rays?
- Are there specific reasons/clinical findings where you wouldn’t refer for X-rays?

**Question 2:** How do you think X-rays help to inform or change your treatment/management of a patient with spinal pain or spinal disorders?

Probes:

- Do X-rays help you reach a diagnosis for the patient? Does this change depending on the clinical case?
- What information do you obtain from X-rays? Does this change depending on the clinical case?
- Do X-rays help you to determine whether a patient needs referral? In what situations?
- Do X-rays help you determine which general management techniques to select (e.g. manipulation, soft tissue techniques, exercise, advice etc.)?
- Do X-rays help you determine how to apply the selected techniques (e.g. change in manipulation type or application)?
- When you receive an X-ray for a patient, how do you generally use it in the clinical consult?
- Do you go through the X-ray with the patient, do you use the X-ray to explain the condition and management to the patient?
- Do you use X-ray findings to change management immediately within a consult?

**Question 3:** In your opinion, does obtaining X-rays generally help to improve your management of a patient or improve the patient outcomes? Why do you feel this?

Probes:

- Are there particular situations where information from X-rays appears to improve patient management or outcomes more than others? Why do you think this is?
- Are there particular situations where information from X-rays does not appear to improve patient management or outcomes? Why do you think this is?

**Question 4:** Is there anything else you would like to add?
