# Supplementary material for: Chiropractors’ perceptions on the use of spinal radiographs in clinical practice: a qualitative study
Source: Chiropr Man Therap. 2024 Jun 22;32:23. doi: 10.1186/s12998-024-00547-y (PMC11193277; doi:10.1186/s12998-024-00547-y)
Supplement: Supplementary file 3 — Supplementary Material 3 [file 12998_2024_547_MOESM3_ESM.docx]

**Manuscript revisions**

**Reviewer #1:** Thanks to the authors for this re-submission. I appreciate how they have handled the comments from both peer reviewers, and for the most part I'm satisfied and think this paper could be moved forward. However, there is still one point that I feel needs to be addressed in more detail. 

1. Limitations - line 452-453. "We did not explicitly address the components of trustworthiness of qualitative data during the design, collection, and analysis of our data."
I apologize, but this response is not sufficient. You need to actually address the components of trustworthiness in qualitative research and comment on how you have or haven't met them. This would be akin to addressing whether your study is valid and reliable in quantitative research and then showing how it is internally and externally valid and meets different applicable aspects of reliability. You have to defend how your findings are actually believable for readers. How are they credible, transferable, dependable, and confirmable?

Once this has been addressed, I will be satisfied. Thanks again and well done.

**Author response:**

We would like to thank reviewer 1 for their comment and appreciate their contribution to improving our manuscript. We believe we have now addressed how we have/ have not addressed the components of trustworthiness.

On page 6-7, line 137 we have included a specific subheading for trustworthiness. Line 138 now reads “Methodological rigour and trustworthiness of the data was addressed throughout the study process (33).”

We included a sentence to explain that our use of an audit trial is an example of dependability/confirmability. Line 142 now reads “To provide dependability and confirmability of the data”.

To address transferability, we have included a sentence from line 145-147. It reads “Transferability of our data was addressed by ensuring we produced a thick description of the participants responses, within their individual contexts by highlighting relevant clinical and demographic characteristics.” Additionally, in our strengths and limitations, we have highlighted that our sufficient breadth and depth of participant characteristics is an example of transferability. Page 21, line 455 now reads “ensuring transferability of our results (43).”

We have now highlighted that we did not perform member checking or peer debriefing during our study, which would have improved the credibility of our results. We have included a paragraph in strengths and limitations to address this. Page 21, line 457-461 now reads “A methodological limitation of this study was not seeking revision of transcripts or results from interview participants (member checking). This practice aims to verify that the results adequately reflect the meaning the participant intended. Additionally, we did not perform peer debriefing with researchers outside of our team. Addressing these aspects of trustworthiness would have strengthened the credibility of our results (44).”
